# 

**Published:** 1889-03

**Authors:** 


VOL. X. MARCH, 1889. No. 3.

THE

### International Dental Journal:

SUCCESSOR TO THE INDEPENDENT PRACTITIONER.

A MONTHLY PERIODICAL

LIBRARY
INDEXED
S.G.O.

DEVOTED TO

### DENTAL AND ORAL SCIENCE.

W. XAVIER SUDDUTH, M.D., D.D.S.,
EDITOR.

PUBLISHED BY

THE INTERNATIONAL DENTAL PUBLICATION COMPANY,

51 WEST 37th STREET, NEW YORK CITY,

—AND—

1215 FILBERT STREET, PHILADELPHIA.

$2.50 Per Annum in Advance, Single Copies, 25 Cents.

